# Zingerone attenuates aortic banding‐induced cardiac remodelling via activating the eNOS/Nrf2 pathway

**DOI:** 10.1111/jcmm.14540

**Published:** 2019-07-10

**Authors:** Chen Liu, Qing-Qing Wu, Zhu-Lan Cai, Sai-Yang Xie, Ming-Xia Duan, Qing-Wen Xie, Yuan Yuan, Wei Deng, Qi‐Zhu Tang

**Affiliations:** ^1^ Department of Cardiology Renmin Hospital of Wuhan University Wuhan China; ^2^ Hubei Key Laboratory of Metabolic and Chronic Diseases Wuhan China

**Keywords:** cardiac remodelling, endothelial nitric oxide synthase, nuclear factor (erythroid‐derived 2)‐like 2, oxidative stress, zingerone

## Abstract

Cardiac remodelling refers to a series of changes in the size, shape, wall thickness and tissue structure of the ventricle because of myocardial injury or increased pressure load. Studies have shown that cardiac remodelling plays a significant role in the development of heart failure. Zingerone, a monomer component extracted from ginger, has been proven to possess various properties including antioxidant, anti‐inflammatory, anticancer and antidiabetic properties. As oxidative stress and inflammation contribute to acute and chronic myocardial injury, we explored the role of zingerone in cardiac remodelling. Mice were subjected to aortic banding (AB) or sham surgery and then received intragastric administration of zingerone or saline for 25 days. In vitro, neonatal rat cardiomyocytes (NRCMs) were treated with zingerone (50 and 250 μmol/L) when challenged with phenylephrine (PE). We observed that zingerone effectively suppressed cardiac hypertrophy, fibrosis, oxidative stress and inflammation. Mechanistically, Zingerone enhanced the nuclear factor (erythroid‐derived 2)‐like 2 (Nrf2)/antioxidant response element (ARE) activation via increasing the phosphorylation of endothelial nitric oxide synthase (eNOS) and nitric oxide (NO) production. Additionally, we used Nrf2‐knockout (KO) and eNOS‐KO mice and found that Nrf2 or eNOS deficiency counteracts these cardioprotective effects of zingerone in vivo. Together, we concluded that zingerone may be a potent treatment for cardiac remodelling that suppresses oxidative stress via the eNOS/Nrf2 pathway.

## 1 INTRODUCTION

Pathological cardiac remodelling is defined by the structural and functional changes in the left ventricle (LV) in response to internal or external cardiovascular damage or influence such as hypertension, myocardial ischaemia, metabolic‐related heart disease and valvular heart disease, and it is a precursor of clinical heart failure (HF).^1^ Accumulating evidence has suggested that the pathological remodelling is associated with fibrosis, inflammation and cellular dysfunction (eg abnormal cardiomyocyte/non‐cardiomyocyte interactions, oxidative stress, autophagy alterations, endoplasmic reticulum (ER) stress and metabolism impairment).^2^ However, our understanding of the key processes responsible for the transition to HF remains incomplete and far less is known about how cardiac remodelling is suppressed.

Oxidative stress refers to the excessive production of reactive oxygen species (ROS) when cells are exposed to harmful stimuli. ROS can directly react with membrane lipids, proteins and nucleic acids to cause apoptosis and necrosis.^3^, ^4^ Additionally, ROS can act as signalling molecules that trigger the production of pro‐inflammatory cytokines.^5^ Accumulating evidence has suggested that oxidative stress is an important factor that exacerbates cardiac remodelling.^6^ Inhibition of oxidative stress could be of significant therapeutic value in the treatment of cardiac remodelling and HF.

Zingerone is a nontoxic and inexpensive compound extracted from a common seasoning in Chinese food, dried ginger, with varied pharmacological activities including antioxidant, anti‐inflammatory, anticancer and antidiabetic activities.^7^ Numerous studies have found that zingerone is a potent antioxidant. It protects DNA against stannous chloride‐induced ROS oxidative damage,^8^ prevents oxidative stress in intestine smooth muscles^9^ and reduces mitochondrial injury and lipid peroxidation.^10^ Furthermore, zingerone has higher antioxidant activity than ascorbic acid^8^ and exerts a scavenging effect against peroxynitrite formed from the reaction of superoxide and nitric oxide.^11^ Recently, accumulating evidence has suggested that zingerone protects the heart from myocardial infarction injury via its antioxidant and free radical scavenging properties.^12^, ^13^, ^14^ Another study revealed that pretreatment with zingerone relieves hyperlipidaemia and cardiac hypertrophy in isoproterenol‐induced myocardial infarcted rats.^15^ However, it is not clear whether zingerone exerts protective effects in AB‐induced cardiac remodelling. Therefore, this investigation was designed to clarify the role of zingerone in cardiac remodelling induced by AB surgery.

## 2 METHODS

### 2.1 Animals and animal models

Male C57/B6J mice were obtained from the Beijing HFK Bioscience CO LTD. eNOS‐knockout (KO) (Stock no. 002684) and Nrf2‐KO (Stock no. 017009) mice were obtained from the Jackson Laboratory. Mice were subject to AB or sham operation as described in the previous study.^16^ Briefly, the thoracic cavity was opened from the second and third ribs on the left side of the mouse, and the thoracic aorta was separated. Then, a 27‐gauge (bodyweight of 24‐25 g) or 26‐gauge (bodyweight of 26‐27 g) needle was placed in the direction parallel to the blood vessel, the blood vessel was ligated with the needle, and the needle was quickly pulled out the of the vessel. Once the blood vessel formed approximately 70% stenosis, the thoracic cavity was closed. In the sham operation group, only a line was hung, but the vessel was not ligated, the rest of the steps were the same as those performed in the surgery group. The male C57/B6 mice were randomly grouped into sham or an AB surgery group with or without zingerone treatment. In the mechanism research section, the Nrf2‐KO and eNOS‐ KO mice were used and were also grouped as before. Four weeks post‐AB or sham procedure, the hearts and lungs of the mice were harvested and weighed. All animal procedures conformed to the National Institutes of Health Guide for the Care and Use of Laboratory Animals (NIH Publications No. 8023, revised 1978) and the Guide for the Care and Use of Laboratory Animals of the Chinese Animal Welfare Committee. The present study was approved by the Animal Use Committees of Renmin Hospital of Wuhan University (approval number: WHRM‐2017W01).

### 2.2 Dosage information

Zingerone was obtained from Winherb Medical Science Co Ltd and was dissolved in sterile saline. Zingerone was intragastrically administered to mice 3 days after AB surgery and repeatedly for another 25 days at a dose of 10 or 20 mg/kg/d according to the previous studies.^17^, ^18^

### 2.3 Echocardiography and haemodynamics

Echocardiography and haemodynamics were performed according to our previous studies.^19^, ^20^ Briefly, a MyLab 30CV ultrasound (Biosound Esaote) with a 10 MHz linear array ultrasound transducer was used for echocardiography measurements. Left ventricular ejection fraction (LVEF), left ventricular fractional shortening (LVFS), left ventricular end‐diastolic diameter (LVEDd), left ventricular end‐systolic diameter (LVESd) and left ventricular end‐diastolic posterior wall dimension (LVPWd) were analysed. Haemodynamics were detected with a microtip catheter transducer (SPR‐839; Millar Instruments). The data of end‐systolic pressure (ESP), end‐diastolic pressure (EDP), minimal rate of pressure decay (dp/dt min) and maximal rate of pressure development (dp/dt max) were recorded and processed by PVAN software.

### 2.4 Histological analysis and immunohistochemistry

The haematoxylin and eosin (HE), Picro Sirius Red (PSR) staining and immunohistochemical staining were performed as previously described.^19^, ^20^ Antibodies against CD68 (Abcam), CD45 (Abcam) and 4‐hydroxynonenal (Abcam) were used for immunohistochemical staining.

### 2.5 Quantitative real‐time RT‐PCR

Total RNA of heart tissues and cells was collected with TRIzol (Invitrogen) and reverse transcribed into cDNA with oligo‐dT primers and reverse transcriptase. Light Cycler 480 SYBR Green 1 Master Mix (Roche) was used for RT‐PCR analysis**.** The transcript quantities were normalized to the amount of GAPDH gene expression.

### 2.6 Western blot

Western blots were performed according to our previous studies.^19^, ^20^ A Nuclear and Cytoplasmic Protein Extraction Kit (Beyotime Institute of Biotechnology) was used for the protein extraction. Primary antibodies against Nrf2 (Abcam), keap1 (Abcam), heme oxygenase‐1 (Abcam), SOD2 (Abcam), NOX2/gp91phox (Abcam), eNOS, eNOS (P‐S1177) (Abcam), GAPDH (Cell Signaling Technology) and lamin B (Abcam) were used. Goat anti‐rabbit IgG (LI‐COR) or goat antimouse IgG (LI‐COR) was used as the secondary antibody.

### 2.7 Cell culture and treatment

Neonatal rat cardiomyocytes were isolated and cultured as our previous study.^21^ PE (50 μmol/L), in the presence or absence of different concentrations (50 μmol/L or 250 μmol/L) of zingerone, was added to the medium, and the cells were incubated for 24 hours. To prevent oxidant stress, we used N‐acetyl‐cysteine (NAC) (Sigma) (2 mmol/L) to treat NRCMs. To inhibit the expression of eNOS or Nrf2, NRCMs were infected with si Nrf 2(20 μmol/L) or si eNOS (50 μmol/L) for 8 hours and then challenged with PE (50 μmol/L) for 24 hours.

### 2.8 ROS detection in NRCMs

Dichlorofluorescein diacetate (DCFH‐DA) was diluted 1:1000 with serum‐free medium to a final concentration of 10 μmol/L. The cell culture medium was removed, and the appropriate volume of diluted DCFH‐DA was added. One well of a six‐well plate received 1 mL of the diluted DCFH‐DA. The plate was incubated for 30 minutes at 37°C in a cell incubator. The cells were washed three times with serum‐free cell culture medium to remove DCFH‐DA that did not enter the cells, and immunofluorescence was observed by a laser confocal microscope.

### 2.9 NO production and SOD activity detection

A Total Nitric Oxide Assay Kit was used to measure NO production (Beyotime Institute of Biotechnology). NO itself is extremely unstable and is rapidly metabolized to nitrate and nitrite in cells, and nitrate reductase is used to reduce nitrate to nitrite; then, the nitrite is detected by the classical Griess reagent to indirectly determine total nitric oxide. The activity of superoxide dismutase (SOD) was detected by a Total Superoxide Dismutase Assay Kit with WST‐8 (Beyotime Institute of Biotechnology) according to the manufacturer's instructions.

### 2.10 Cell viability assay

The cells were incubated in a 96‐well plate. After the cells were attached, different concentrations of zingerone were added to each well and incubated for 24 hours at 37°C. Then, MTT (5 mg/mL) was added and incubated for 4 hours. The culture solution was discarded, 100 µL of DMSO was added to each well, and the absorbance value was measured after 10‐minutes incubation.

### 2.11 Immunofluorescence

The cells were incubated in a 24‐well plate; after the cells were attached, the medium was discarded, 4% paraformaldehyde was added to fix the cells, and 0.1% Triton X‐100 was added to permeabilize them. Primary α‐actinin (Abcam) and Nrf2 (Abcam) antibodies were applied at 4°C overnight; goat anti‐rabbit (LI‐COR) was used as the secondary antibody and was added to the cells and incubated for 60 minutes at 37°C. Immunofluorescence was observed by a laser confocal microscope.

### 2.12 Data and statistical analysis

The data are expressed as the means ± SD and were analysed by SPSS software, version 23.0. One‐way ANOVA followed by Tukey's post hoc test was used for group comparisons. Two‐group comparisons were performed by Student's unpaired *t* test. The value of *P* < .05 was defined as significantly different.

## 3 RESULT

### 3.1 Zingerone suppressed PE‐induced NRCMs hypertrophy

**Figure 1:**
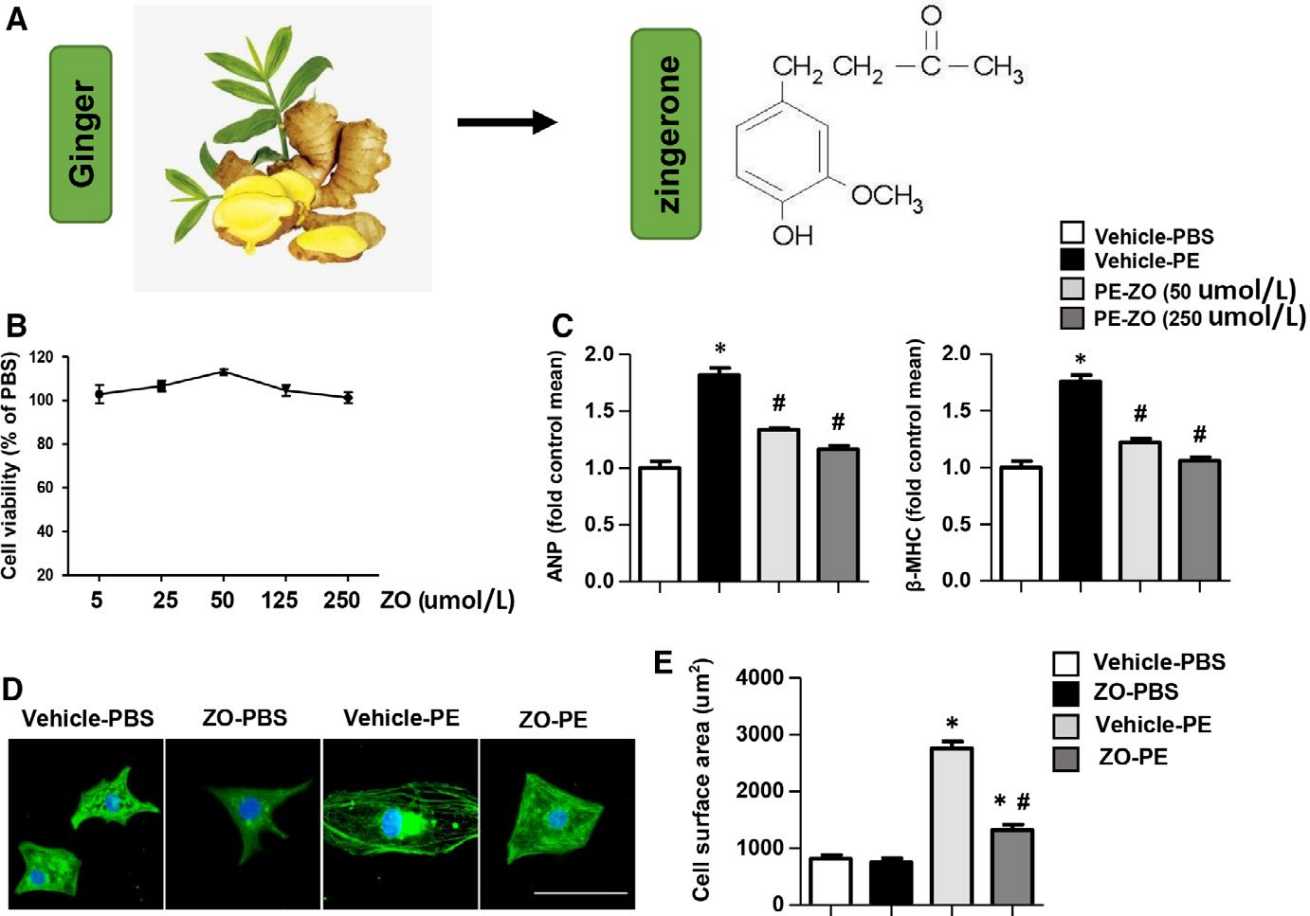
Zingerone suppresses PE‐induced NRCM hypertrophy. A, An image of ginger and the chemical structure of zingerone. B, NRCMs were treated with zingerone (5, 25, 50, 125 and 250 μmol/L) for 24 h. The cell viability was detected by MTT assays. (C) NRCMs were treated with zingerone (50 and 250 μmol/L) and PE (50 μmol/L) for 24 h. RT‐PCR analysis was performed to detect the ANP and β‐MHC mRNA levels in each group (n = 6 samples). (D and E) NRCMs were treated with zingerone (250 μmol/L) and PE (50 μmol/L) for 24 h. Representative images (D) and quantitative results (E) of α‐actinin immunofluorescence staining in each group (n = 6 samples, 50 + cells per group). *P < .05 vs the vehicle‐PBS group; #*P* < .05 vs the vehicle‐PE group

To investigate the effects of zingerone on cardiac hypertrophy, we treated NRCMs with a hypertrophic agonist, PE (50 μmol/L), and assessed the effect of zingerone on NRCM hypertrophy. First, we determined different zingerone dose‐responses in NRCMs. As shown in Figure 1B, the cell toxicity of zingerone was not significantly different under the concentrations of 5, 25, 50, 125 and 250 μmol/L. The effective concentration of 50 μmol/L and 250 μmol/L zingerone significantly decreases atrial natriuretic peptide (ANP) and β‐myosin heavy chain (β‐MHC) mRNA expression levels with PE challenge (Figure 1C). The cell surface area of NRCMs was also both decreased at the concentration of 250 μmol/L zingerone treatment (Figure 1D‐E).

### 3.2 Zingerone prevented cardiac hypertrophy and fibrosis induced by AB surgery

**Figure 2:**
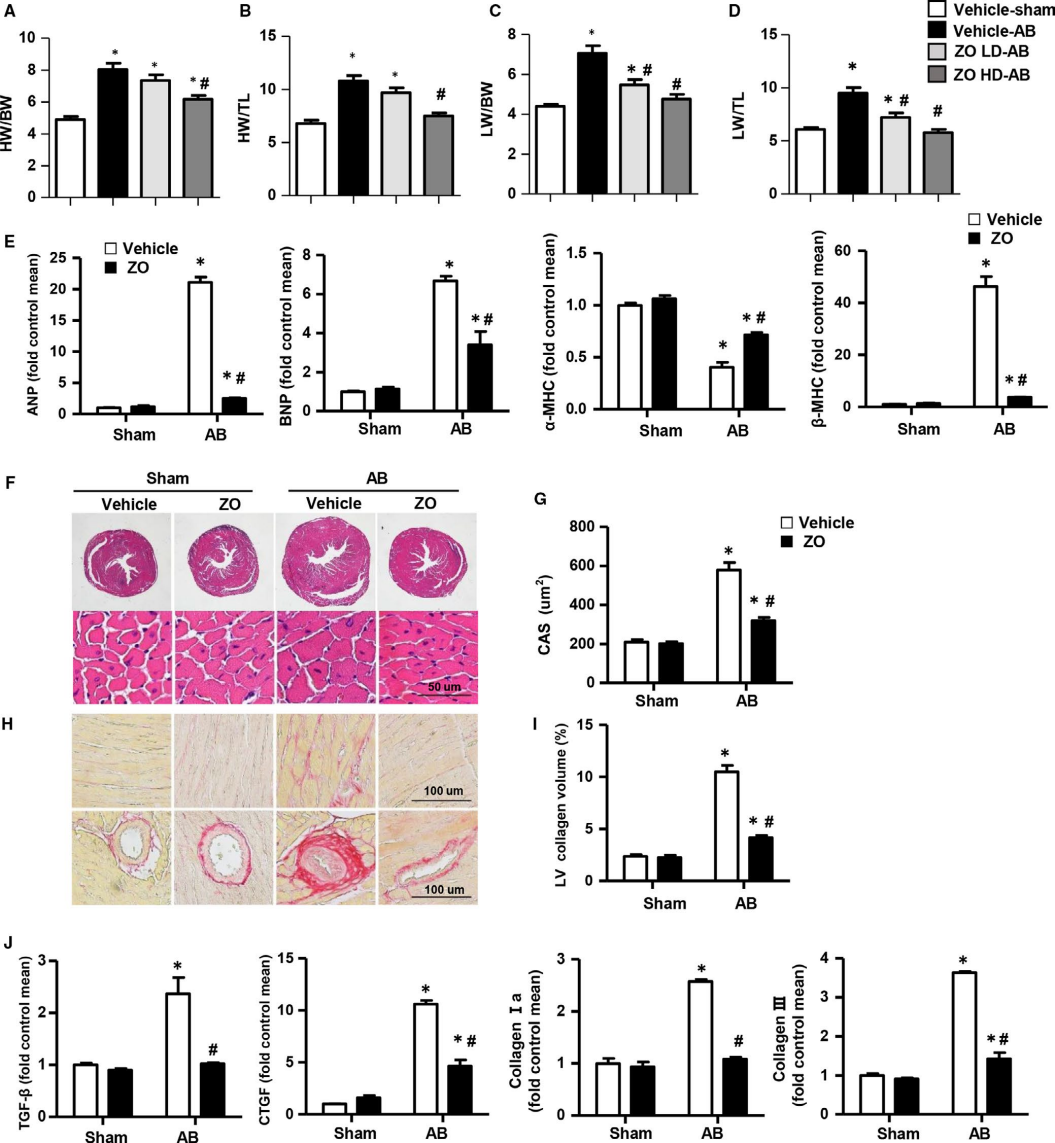
Zingerone prevented cardiac hypertrophy and fibrosis induced by AB surgery. (A‐D) The ratios of HW/BW, HW/TL, LW/BW and LW/TL in zingerone‐ and vehicle‐treated mice after AB or sham surgery (n = 8, low dose [LD]: 10 mg/kg/d; high dose [HD]: 20 mg/kg/d). E, RT‐PCR analyses of hypertrophic markers (ANP, BNP, β‐MHC, α‐MHC) in each group (n = 6). (F and G) HE staining and CAS results of zingerone‐treated and vehicle‐treated mice after AB or sham surgery (n = 6, 100 + cells per group). Representative images (H) and quantification of the total collagen volume (I) of PSR staining in the indicated groups (n = 6, 10 + fields per heart). J, RT‐PCR analyses of fibrotic markers (collagen I, collagen III, TGFβ and CTGF) in each group (n = 6). *P < .05 vs vehicle‐sham; #*P* < .05 vs vehicle‐AB

To explore the effects of zingerone on cardiac remodelling, all mice were subjected to AB surgery or a sham operation with or without 25 days of zingerone intragastric administration at 3 days post‐surgery. The ratios of heart weight/bodyweight (HW/BW) (mg/g), heart weight/tibia length (HW/TL) (mg/mm), lung weight/bodyweight (LW/BW) (mg/g) and lung weight/tibia length (LW/TL) (mg/mm) were significantly decreased in mice treated with the high dose of zingerone (20 mg/kg/d) after AB compared with the vehicle‐treated mice after AB, while the HW/BW and HW/TL ratios in the mice treated with the low dose of zingerone (10 mg/kg/d) after AB were not significantly decreased (Figure 2A‐D). Thus, 20 mg/kg/d zingerone was used for further study. The cross‐sectional area (CAS) and mRNA levels of hypertrophic markers, ANP, B‐type natriuretic peptide (BNP) and β‐MHC were increased, and a‐myosin heavy chain (a‐MHC) was decreased in vehicle‐treated mice after AB. These alterations were severely blunted in the zingerone‐treated mice after AB surgery (Figure 2E‐G). PSR staining was used for the detection of perivascular and interstitial fibrosis. As shown in Figure 2H‐I, the degree of perivascular and interstitial fibrosis was remarkably reduced in zingerone‐treated mice subjected to AB. Additionally, the mRNA levels of fibrosis markers, transforming growth factor β1 (TGF‐β1), connective tissue growth factor (CTGF), and collagen I and collagen III were dramatically decreased in zingerone‐treated mice subjected to AB (Figure 2J). These results indicated that zingerone abated the cardiac hypertrophy and fibrosis induced by AB surgery.

### 3.3 Zingerone relieved pressure overload‐induced heart dysfunction in mice

As shown in Table S1 in Appendix S1, vehicle‐treated mice after AB surgery exhibited aggravated cardiac function, with reduced LVEF, LVFS and dilated left ventricular diameter (increased LVEDd, LVESd) and wall hypertrophy (increased LVPWd). However, the cardiac dysfunction was alleviated dramatically in zingerone‐treated mice after AB, which was assessed by the elevated LVEF and LVFS and reduced LV diameter and wall thickness. Additionally, haemodynamic parameters also displayed better systolic and diastolic LV functions in zingerone‐treated mice after AB, as assessed by ESP, EDP, dp/dt min and dp/dt max. Accordingly, zingerone administration could alleviate heart dysfunction in mice after AB surgery.

### 3.4 Zingerone attenuated cardiac oxidative stress and inflammation in vivo

**Figure 3:**
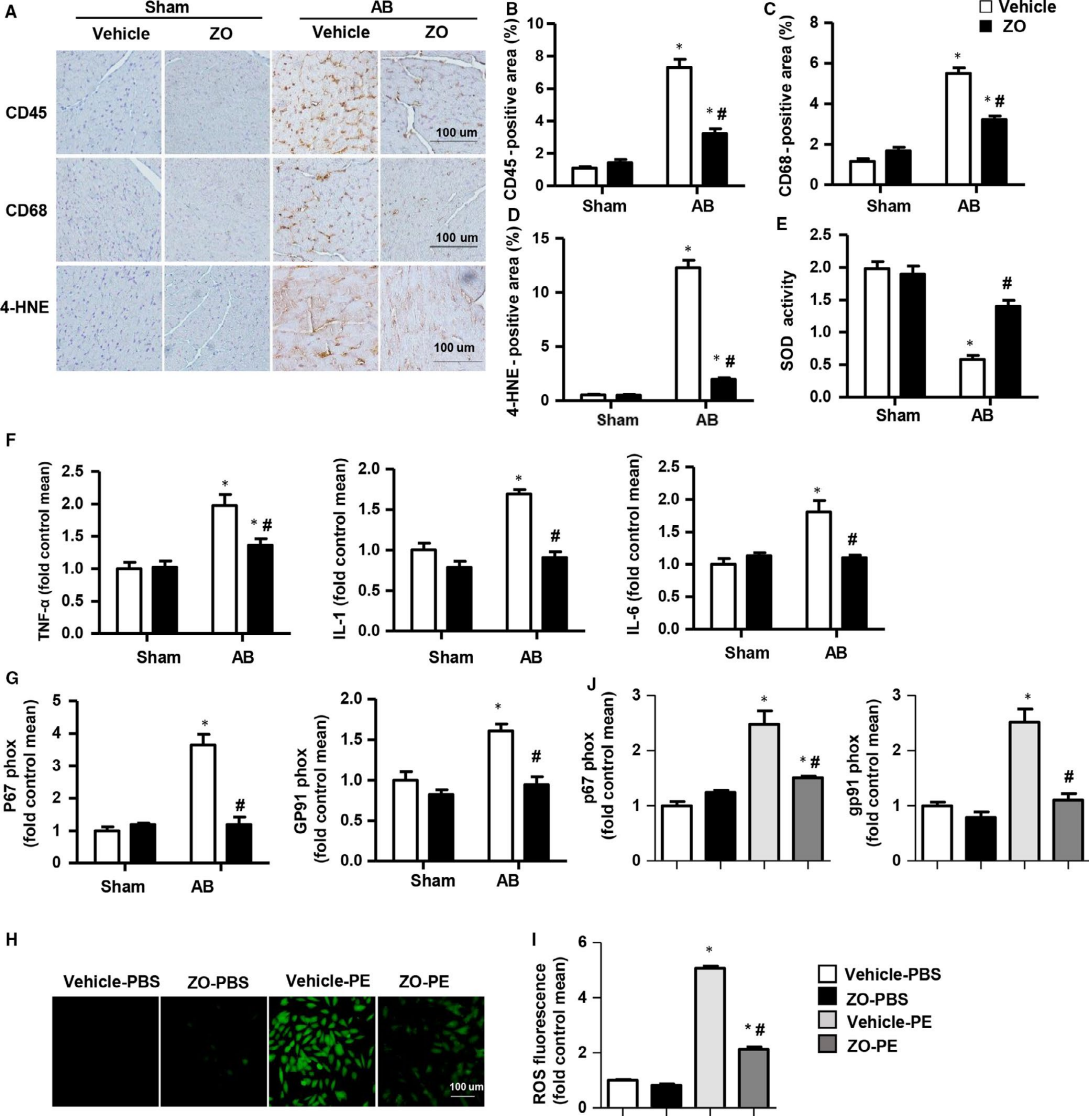
Zingerone attenuated cardiac oxidative stress and inflammation in vivo and in vitro. A, Immunohistochemical staining of CD45, CD68 and 4‐HNE in mouse hearts in each group. B‐D, Quantification of CD45, CD68 and 4‐HNE‐positive cells in mouse hearts in each group (n = 6, 10 + fields per heart). E, SOD activation in each group (n = 6). F, RT‐PCR analyses of pro‐inflammatory cytokines (TNF‐α, IL‐1β, IL‐6) in each group (n = 6). G, RT‐PCR analyses of oxidative markers (NADPH gp91 phox, NADPH p67 phox) in each group (n = 6). *P < .05 vs vehicle‐sham; #*P* < .05 vs vehicle‐AB. H‐J, NRCMs were treated with zingerone (250 μmol/L) and were stimulated with PE (50 μmol/L) for 24 h. H, ROS detection was performed by DCFH‐DA (n = 6 samples). I, Quantitative results of ROS fluorescence. J, RT‐PCR analyses of oxidative markers (NADPH gp91 phox and NADPH p67 phox) in each group (n = 6 samples). *P < .05 vs the vehicle‐PBS group; #*P* < .05 vs the vehicle‐PE group

Because of the potent antioxidant and anti‐inflammatory properties of zingerone, we further investigated the effect of zingerone on cardiac oxidative stress and inflammation, and we found that the CD45‐labelled leucocytes, CD68‐labelled macrophage infiltration and 4‐hydroxynonenal (4‐HNE) production decreased and that the activity of SOD increased in the hearts of the zingerone‐treated mice subjected to AB (Figure 3A‐E). The mRNA expression levels of the pro‐inflammatory cytokines(interleukin‐1 [IL‐1], interleukin‐6 [IL‐6] and tumour necrosis factor alpha [TNF‐α]) and of NADPH oxidase (NADPH P67 phox and NADPH GP91 phox) were dramatically decreased in zingerone‐treated mice after AB surgery compared with those of the vehicle‐AB group (Figure 3F‐G).

### 3.5 Zingerone attenuated oxidative stress in vitro

In vitro, we detected the cellular ROS generation in each group, and we found that zingerone treatment dramatically inhibited PE‐induced ROS generation and decreased the mRNA levels of NADPH P67 phox and GP91 phox (Figure 3H‐J). The ROS scavenger NAC was used to determine whether the protective effects of zingerone were based on its antioxidative properties. As a result, NAC suppressed the PE‐induced cardiac hypertrophy, and zingerone (250 μmol/L) could not augment this improvement, as demonstrated by the same extent of reduction in the transcription levels of hypertrophic markers and ROS generation between the PE + NAC group and the NAC + PE+zingerone group (Figure S1A‐E in Appendix S1).

### 3.6 Zingerone enhanced Nrf2/ARE activation in vivo and in vitro

**Figure 4:**
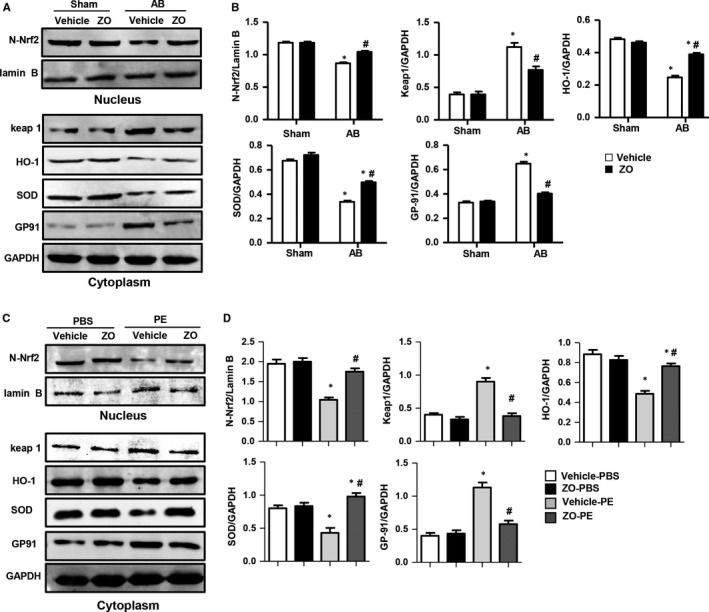
Zingerone enhanced the Nrf2/ARE pathway activation in vivo and in vitro. Western blot image (A) and quantitative results (B) of the Nrf2 pathway of each group in vivo (n = 6). *P < .05 vs vehicle‐sham; #*P* < .05 vs vehicle‐AB. (C and D) NRCMs were treated with zingerone (250 μmol/L) and PE (PE 50 μmol/L) for 24 h. Western blot images (C) and quantitative results (D) of the Nrf2 pathway in each group (n = 6 samples). *P < .05 vs the vehicle‐PBS group; #*P* < .05 vs the vehicle‐PE group

**Figure 5:**
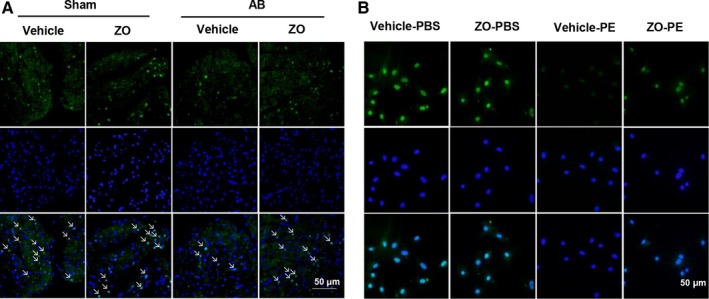
Zingerone enhanced the Nrf2 nuclear translocation in vivo and in vitro. A, Immunofluorescence staining of Nrf 2 was used to detect the Nrf2 nuclear translocation on myocardial sections (n = 6 samples). B, Immunofluorescence staining of Nrf 2 was used to detect the Nrf2 nuclear translocation on NRCMs (n = 6 samples, 50 + cells per group)

Nrf2 is a defensive pathway involved in oxidative and chemical stress. We next explored whether zingerone affected the activation of Nrf2. We found decreased protein expression levels of nucleus‐Nrf2 (N‐Nrf2), heme oxygenase‐1 (HO‐1) and SOD and increased protein expression levels of Kelch‐like ECH‐associated protein (keap1) and NOX2/gp91 phox after AB surgery in vivo and after PE challenge in vitro. Zingerone treatment enhanced Nrf2 activation with increased N‐Nrf2, HO‐1 and SOD, and decreased keap1 and NOX2/gp91 phox protein expression levels after AB surgery in vivo (Figure 4A‐B) and after PE challenge in vitro (Figure 4C‐D) Additionally, the immunostaining for Nrf2 on myocardial sections and NRCMs also confirmed the translocation of Nrf2 into the nucleus in the zingerone + AB and zingerone + PE groups in vivo and in vitro. (Figure 5A‐B).

### 3.7 The effects of Nrf2 in zingerone‐mediated cardioprotection in vivo and in vitro

**Figure 6:**
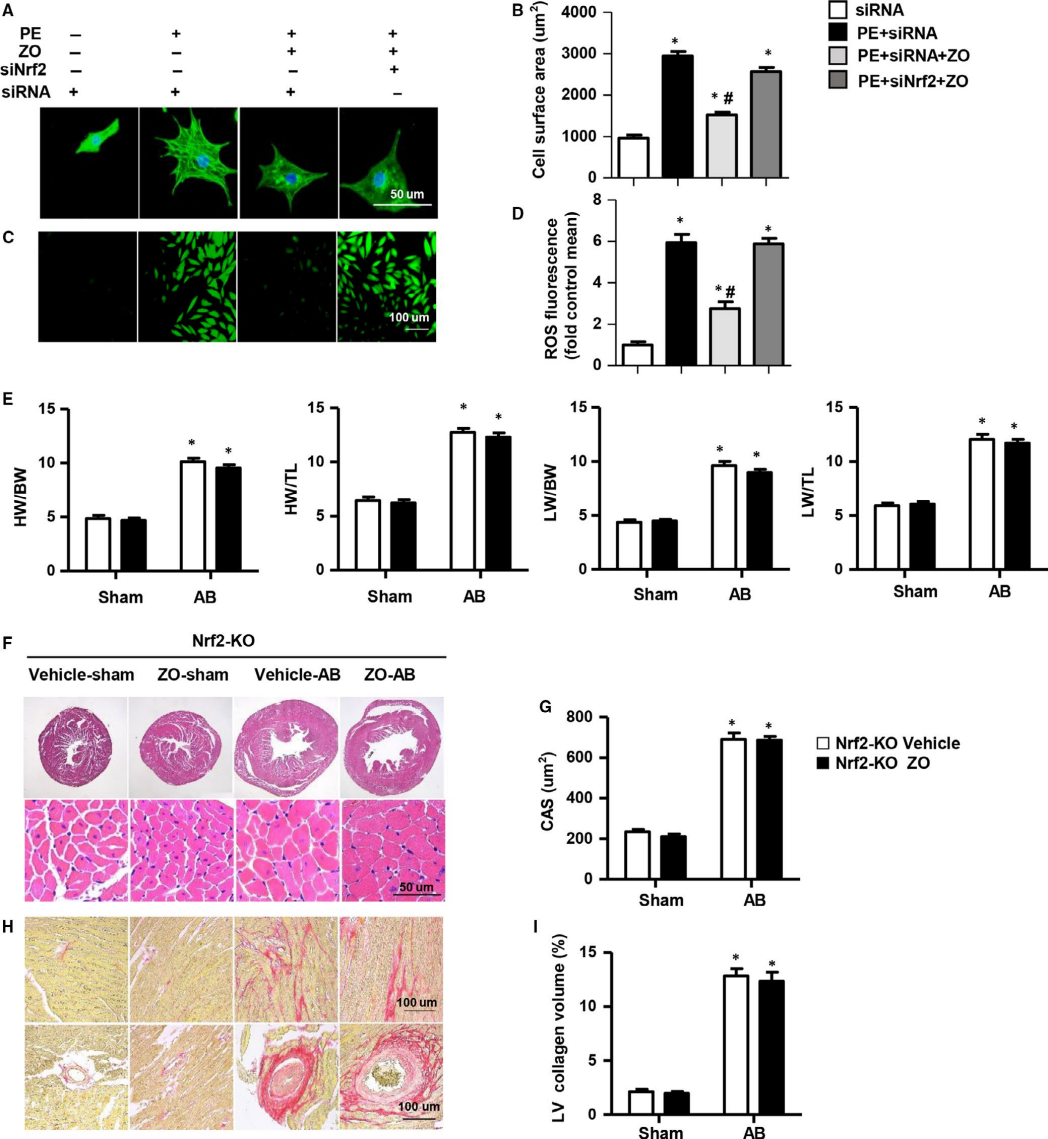
The effects of Nrf2 in zingerone‐mediated cardioprotection in vivo and in vitro. A‐D, NRCMs were treated with zingerone (250 μmol/L) and/or siNrf2 (20 μmol/L) and were stimulated with PE (50 μmol/L) for 24 h. A‐B, The cell surface area was detected by immunofluorescence staining of α‐actinin (n = 6 samples, 50 + cells per group). A, Representative images; B, quantitative results. C, Representative images of ROS detection (n = 6 samples). D, Quantitative results of ROS fluorescence. *P < .05 vs the siRNA group; #*P* < .05 vs the PE + siRNA group. E, The ratios of HW/BW, HW/TL, LW/BW and LW/TL in zingerone‐ or vehicle‐treated Nrf2‐KO mice (n = 8). (F and G) Haematoxylin and eosin staining in each group (n = 6). F, Representative images; (G) the analysis of CSA. (H and I) Representative images and quantification of the total collagen volume of PSR staining in each group (n = 6). *P < .05 vs Nrf2‐KO vehicle‐sham; #*P* < .05 vs Nrf2‐KO vehicle‐AB

To further evaluate the necessity of the Nrf2 pathway on the anti‐remodelling effects of zingerone, NRCMs were treated with si Nrf2 to knock‐down Nrf2 (Figure S2A‐B in Appendix S1). Interestingly, zingerone‐mediated cardioprotective effects (anti‐hypertrophy and antioxidative stress) were counteracted by Nrf2 knock‐down (Figure 6A‐D). Furthermore, the transcription levels of the hypertrophic markers and NADPH oxidase such as ANP, β‐MHC, P67 phox and GP91 phox were elevated after Nrf2 knock‐down (Figure S2E in Appendix S1). In vivo, we used Nrf 2‐KO mice and found that the HW/BW, LW/BW, HW/TL and LW/TL ratios increased in both the zingerone‐treated and vehicle‐treated mice after AB surgery (Figure 6E). Additionally, the heart function (Table S2 in Appendix S1), cardiomyocyte area (Figure 6F‐G), LV collagen volume (Figure 6H‐I) and transcription level of hypertrophic and fibrotic markers showed no significant differences between the zingerone‐ and vehicle‐treated mice after AB (Figure S3A‐B in Appendix S1). These results indicated that Nrf2 deletion eliminated the cardioprotection of zingerone treatment.

### 3.8 Zingerone improved the eNOS activation and NO production in vivo and in vitro

**Figure 7:**
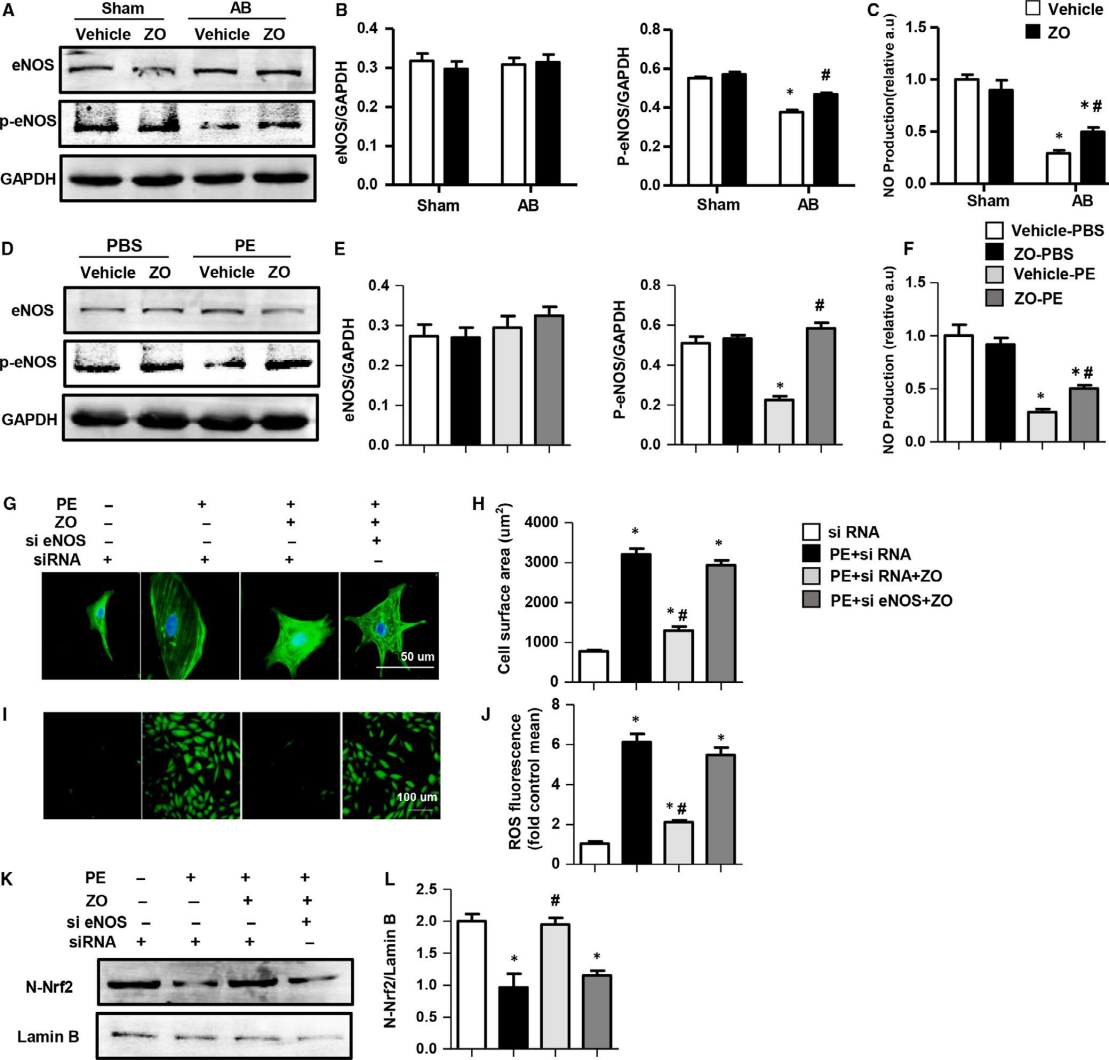
Zingerone improved eNOS activation and NO production in vivo and in vitro and the effects of eNOS on zingerone‐mediated cardioprotection in vitro. Western blot image (A) and quantitative results (B) of eNOS and p‐eNOS in each group in vivo (n = 6). C, NO production in vivo (n = 6). *P < .05 vs vehicle‐sham; #*P* < .05 vs vehicle‐AB. D‐F, NRCMs were treated with zingerone (250 μmol/L) and PE (PE 50 μmol/L) for 24 h. Western blot images (D) and quantitative results (E) of eNOS and p‐eNOS in each group in vitro (n = 6 samples). F, NO production in vitro (n = 6 samples). *P < .05 vs the vehicle‐PBS group; #*P* < .05 vs the vehicle‐PE group. G‐K, NRCMs were treated with zingerone (250 μmol/L) and/or si eNOS (20 μmol/L) and were stimulated with PE (50 μmol/L) for 24 h. Representative images (G) and quantitative results (H) of cell surface areas detected by α‐actinin immunofluorescence staining in each group (n = 6 samples, 50 + cells per group). I, Representative images of ROS detection (n = 6 samples). J, Quantitative results of ROS fluorescence. Western blot image (K) and quantitative results (L) of N‐Nrf2 in each group (n = 6 samples). *P < .05 vs the siRNA group; #*P* < .05 vs the siRNA + PE group

It is well‐established that the transcriptional regulation of those antioxidant genes by Nrf2 signalling is mainly through NO.^22^ The biosynthesis of NO by NOS enzymes is well defined. Additionally, increased phosphorylation at Ser1177/9 leads to eNOS activation and to elevated NO production.^23^ To further evaluate the mechanism underlying how zingerone affects the Nrf2 pathway, we next investigated the eNOS‐NO axis. We found that the eNOS expression levels were unchanged, but p‐eNOS (Ser1177) expression levels were reduced in mouse heart after AB surgery in vivo and in NRCMs with PE challenge in vitro. Zingerone treatment increased the p‐eNOS level after AB surgery in vivo and after PE challenge in vitro. Moreover, the NO production increased accompanied with the eNOS phosphorylation both in vivo and in vitro (Figure 7A‐F).

### 3.9 Zingerone‐mediated cardioprotection depends on the eNOS/Nrf2 pathway in vitro

To further evaluate the effects of eNOS on zingerone‐mediated cardioprotection, we treated the NRCMs with si eNOS (Figure S2C‐D in Appendix S1). As expected, zingerone‐mediated cardioprotective effects (anti‐hypertrophy and antioxidative stress) in NRCMs were effectively abolished by silencing eNOS (Figure 7G‐J, Figure S2F in Appendix S1). Moreover, the protein expression of N‐Nrf2 was significantly decreased after PE + si RNA treatment and dramatically increased with PE + si RNA + zingerone treatment; however, after eNOS knock‐down, the N‐Nrf2 protein expression was significantly down‐regulated (Figure 7K‐L). Additionally, Nrf2 silencing did not influence the expression of eNOS, p‐eNOS (S1177) and NO production in NRCMs (Figure S4A‐C in Appendix S1).

### 3.10 eNOS deficiency counteracted the cardioprotection effects of Zingerone in vivo

**Figure 8:**
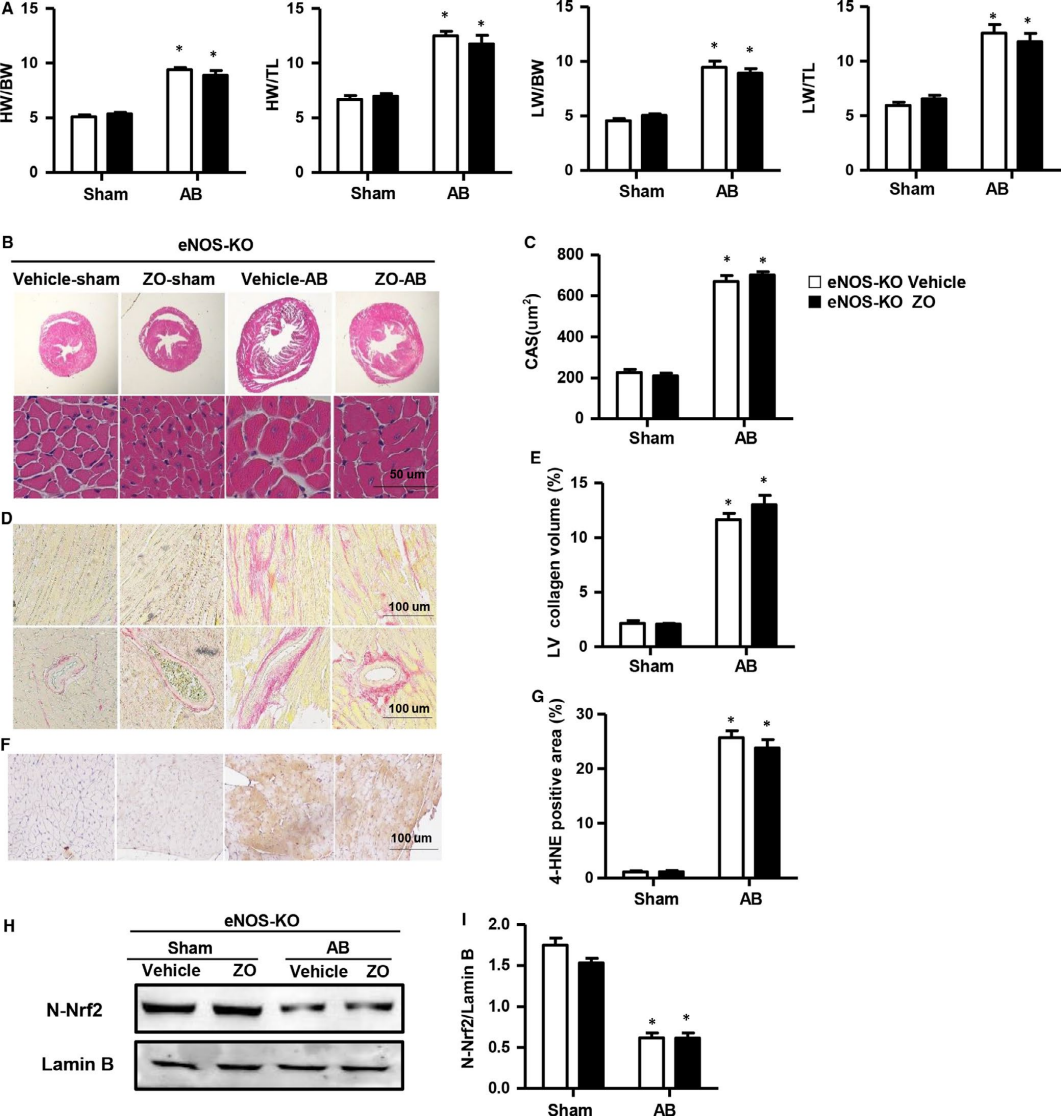
eNOS deficiency counteracts the protective effects of zingerone in vivo. A, The ratios of HW/BW, HW/TL, LW/BW and LW/TL in zingerone‐ or vehicle‐treated eNOS‐KO mice (n = 8). (B and C) Haematoxylin and eosin staining in each group (n = 6). B, Representative images; (C) the analysis of CSA. (D and E) Representative images and quantification of the total collagen volume of PSR staining in each group (n = 6). F, Immunohistochemical staining of 4‐HNE in each group (n = 6, 10 + fields per heart). G, Quantification of 4‐HNE‐positive cells in mouse hearts in each group. Western blot image (H) and quantitative results (I) of N‐Nrf2 in each group (n = 6). *P < .05 vs eNOS‐KO vehicle‐sham; #*P* < .05 vs eNOS‐KO vehicle‐AB

In vivo, we further used eNOS‐KO mice to investigate the effect of eNOS on zingerone‐mediated cardioprotection. Consistent with the results in vitro, the cardioprotection of zingerone treatment after AB surgery was effectively abolished by eNOS deletion, as demonstrated by the same increase in the HW/BW, LW/BW, HW/TL and LW/TL ratios between zingerone‐treated and vehicle‐treated mice after AB surgery (Figure 8A). In addition, the heart function (Table S3 in Appendix S1), cardiomyocyte area (Figure 8B‐C), LV collagen volume (Figure 8D‐E), 4‐HNE production (Figure 8F‐G) and transcription level of hypertrophic, fibrotic and oxidative stress markers showed no significant differences between the zingerone‐ and vehicle‐treated mice after AB (Figure S3C‐E in Appendix S1). Additionally, zingerone treatment did not enhance Nrf2 activation because of the same decreased N‐Nrf2 expression in zingerone‐treated and vehicle‐treated mice after the AB surgery (Figure 8H‐I). These data suggested that eNOS ablation completely eliminated the cardioprotection of zingerone treatment. And that zingerone‐mediated cardioprotection depends on the eNOS/Nrf2 pathway.

## 4 DISCUSSION

Ginger is one of the most common spices originating in South‐East Asia. Zingerone is present in significant amounts (9.25%) in ginger, and cooking or drying also converts gingerol (another component in ginger) into zingerone by a retro‐aldol reaction. Zingerone is known to have potent pharmacological activities, such as antioxidant and anti‐inflammatory properties.^7^ Numerous studies have suggested that zingerone protects against cardiac oxidative stress and inflammation damage in the streptozotocin‐induced diabetic mice,^24^ isoproterenol‐induced myocardial infarction rats,^13^, ^14^ chemo/radiotherapy‐mediated cardiac damage, among others.^25^ These data suggest a potential cardioprotection of zingerone in heart diseases, especially in cardiac remodelling. Our research further explored its effect in pressure overload‐induced cardiac remodelling and found that zingerone significantly inhibited pathological cardiac hypertrophy, fibrosis, heart dysfunction, inflammation and oxidative stress induced by hypertrophic stimuli. Furthermore, we also revealed that the mechanism by which zingerone inhibits oxidative stress following hypertrophic stress is through the eNOS‐NO‐Nrf2 signalling pathway.

Oxidative stress is generated when ROS overwhelm the antioxidant enzymes. To combat the harm caused by oxidative stress, cells defend themselves with enzymes such as SOD, catalase and glutathione peroxidase.^26^ There is overwhelming evidence indicating that increased oxidative stress and reduced activity of antioxidants are closely related to the development and propagation of cardiac hypertrophy and HF in animal models and humans.^5^, ^6^ Nrf2 is a redox‐sensitive transcription factor that normally resides in the cytoplasm bound to keap1. Upon exposure to pro‐oxidative or electrophilic stimuli, cysteine residues of keap1 are oxidized or covalently modified, and Nrf2 is released to the nucleus. By binding to ARE consensus sequences, Nrf2 initiates many antioxidant genes that preserve cellular homeostasis.^27^, ^28^ In the mechanism of zingerone‐mediated cardioprotection against pathological cardiac remodelling, we found a dramatic inhibition of oxidative stress by zingerone, and the effect of the antioxidant NAC on reducing NRCM hypertrophy was not further improved by zingerone. Furthermore, the Nrf2 pathway was enhanced both in vivo and in vitro after zingerone treatment to eliminate the excessive oxidative stress. Thus, zingerone could augment the Nrf2 pathway to suppress oxidative stress and to exert a cardioprotective effect in cardiac remodelling. ROS could also act as signalling molecules to trigger pro‐inflammatory cytokine production.^26^ In our investigation, cardiac inflammation was also inhibited by zingerone.

The free radical nitric oxide (NO) is one of the most widespread signalling molecules and is synthesized from the oxidation of L‐arginine by NOS enzymes. eNOS is the main contributor of circulating NO. The activity of eNOS can be initiated in both calcium‐dependent and calcium‐independent manners.^29^ Phosphorylation of eNOS independently of the calcium concentration is also significant for eNOS activation. However, different phosphorylation sites of eNOS can have opposing effects,^23^ Ser1177 (or Ser1179 depending on the species) is an activation site, whereas Thr495 is an inhibitory site. Previous research showed that protein kinase B (AKT) and AMP‐activated protein kinase (AMPK) could phosphorylate eNOS at the Ser1177 site in response to various stimuli.^30^, ^31^ In our study, p‐eNOS (Ser1177) expression dramatically increased with zingerone treatment after AB surgery in vivo and after PE challenge in vitro, thus leading to increased production of NO.

Accumulating evidence indicates the importance of NO in heart diseases with a spectrum of related pathologies, which include hypertension,^32^ CVD^33^ and atherogenesis.^34^ It is well known that NO is involved in the activation of Nrf2. NO activates Nrf2 translocation into the nucleus from the cytoplasm and targets antioxidant responsive element (ARE), directly increasing the transcription of many antioxidant enzymes including glutathione S‐transferase (GST), glutathione peroxidase 1 (GPx‐1), NAD(P)H quinone oxidoreductase 1 (NQO1), SOD and HO‐1.^35^ Tarak Nath Khatua et al^36^ found that activation of the eNOS‐Nrf2‐Tfam pathway could induce mitochondrial biogenesis and ameliorate isoproterenol‐induced cardiac remodelling in rats. Ruimin Wang et al^37^ indicated that enhanced eNOS activation and (NO)‐mediated thiol modification of keap1, with subsequent up‐regulation of the Nrf2/HO‐1 antioxidative signalling pathway, exert profound neuroprotection, antioxidant and cognitive function preservation effects in global cerebral ischaemia (GCI). During our investigation, zingerone increased NO production, which enhanced Nrf2 translocation into the nucleus and increased the expression of the antioxidant enzymes SOD and HO‐1, which limited the remodelling response. However, some investigations have demonstrated regulation of the eNOS activity and Nrf2‐mediated gene expression that were contrary to our findings. Heiss et al's investigation^38^ indicated that activated Nrf2 contributed to eNOS coupling by ensuring stoichiometric balance between BH_4_ and eNOS and that it enhanced the bioavailability of NO but transiently decreased eNOS levels in primary endothelial cells. The recent study of Erkens et al^39^ found that Nrf2 deficiency, despite decreased antioxidant reserve capacity, up‐regulated eNOS expression and exerted cardioprotective effects against I/R injury in 5‐ to 6‐month‐old ageing male mice. The effect may be attributed to variations in the regulation between Nrf2 and NO or eNOS in different cells, different disease models, different tissues and even different ages of mice.

Based on the available information, it is apparent that zingerone promotes eNOS activation, increasing NO production and targeting Nrf2‐associated antioxidant gene transcription in cardiac remodelling. Therefore, zingerone may be considered of therapeutic interest in the prevention and treatment of cardiac remodelling and HF.

## CONFLICT OF INTEREST

The authors declare no conflict of interest.

## AUTHOR CONTRIBUTION

Chen Liu, Qing Qing Wu and Qi‐Zhu Tang contributed to the conception and design of the experiments; Chen Liu, Zhulan Cai, Saiyang Xie, Mingxia Duan and Qinwen Xie carried out the experiments; Chen Liu, Qing Qing Wu, Yuan Yuan and Wei Deng analysed the data. Chen Liu wrote the manuscript; Chen Liu and Qing Qing Wu revised the manuscript.

## Data Availability

The data will be made available after been required upon request from the corresponding author.

## References

[1] Wu QQ , Xiao Y , Yuan Y , et al. Mechanisms contributing to cardiac remodelling. Clin Sci. 2017;131:2319‐2345. 28842527 10.1042/CS20171167

[2] Kehat I , Molkentin JD . Molecular pathways underlying cardiac remodeling during pathophysiological stimulation. Circulation. 2010;122:2727‐2735. 21173361 10.1161/CIRCULATIONAHA.110.942268 PMC3076218

[3] Poprac P , Jomova K , Simunkova M , Kollar V , Rhodes CJ , Valko M . Targeting free radicals in oxidative stress‐related human diseases. Trends Pharmacol Sci. 2017;38:592‐607. 28551354 10.1016/j.tips.2017.04.005

[4] Santilli F , D'Ardes D , Davi G . Oxidative stress in chronic vascular disease: from prediction to prevention. Vascul Pharmacol. 2015;74:23‐37. 26363473 10.1016/j.vph.2015.09.003

[5] Ayoub KF , Pothineni N , Rutland J , Ding Z , Mehta JL . Immunity, inflammation, and oxidative stress in heart failure: emerging molecular targets. Cardiovasc Drugs Ther. 2017;31:593‐608. 28956198 10.1007/s10557-017-6752-z

[6] Maulik SK , Kumar S . Oxidative stress and cardiac hypertrophy: a review. Toxicol Mech Methods. 2012;22:359‐366. 22394344 10.3109/15376516.2012.666650

[7] Ahmad B , Rehman MU , Amin I , et al. A review on pharmacological properties of zingerone (4‐(4‐Hydroxy‐3‐methoxyphenyl)‐2‐butanone). ScientificWorldJournal. 2015;2015:816364. 26106644 10.1155/2015/816364 PMC4461790

[8] Rajan I , Narayanan N , Rabindran R , Jayasree PR , Manish Kumar PR . Zingerone protects against stannous chloride‐induced and hydrogen peroxide‐induced oxidative DNA damage in vitro. Biol Trace Elem Res. 2013;155:455‐459. 24006104 10.1007/s12011-013-9801-x

[9] Banji D , Banji OJ , Pavani B , Kranthi Kumar C , Annamalai AR . Zingerone regulates intestinal transit, attenuates behavioral and oxidative perturbations in irritable bowel disorder in rats. Phytomedicine. 2014;21:423‐429. 24262066 10.1016/j.phymed.2013.10.007

[10] Vaibhav K , Shrivastava P , Tabassum R , et al. Delayed administration of zingerone mitigates the behavioral and histological alteration via repression of oxidative stress and intrinsic programmed cell death in focal transient ischemic rats. Pharmacol Biochem Behav. 2013;113:53‐62. 24141173 10.1016/j.pbb.2013.10.008

[11] Shin SG , Kim JY , Chung HY , Jeong JC . Zingerone as an antioxidant against peroxynitrite. J Agric Food Chem. 2005;53:7617‐7622. 16159194 10.1021/jf051014x

[12] Stanely Mainzen Prince P , Hemalatha KL . A molecular mechanism on the antiapoptotic effects of zingerone in isoproterenol induced myocardial infarcted rats. Eur J Pharmacol. 2018;821:105‐111. 28982542 10.1016/j.ejphar.2017.09.051

[13] Hemalatha KL , Stanely Mainzen Prince P . A biochemical and 2, 3, 5‐triphenyl tetrazolium chloride staining study on the preventive effects of zingerone (vanillyl acetone) in experimentally induced myocardial infarcted rats. Eur J Pharmacol. 2015;746:198‐205. 25445034 10.1016/j.ejphar.2014.10.057

[14] Hemalatha KL , Prince PS . Preventive effects of zingerone on altered lipid peroxides and nonenzymatic antioxidants in the circulation of isoproterenol‐induced myocardial infarcted rats. J Biochem Mol Toxicol. 2015;29:63‐69. 25271244 10.1002/jbt.21668

[15] Hemalatha KL , Stanely Mainzen Prince P . Antihyperlipidaemic, antihypertrophic, and reducing effects of zingerone on experimentally induced myocardial infarcted rats. J Biochem Mol Toxicol. 2015;29:182‐188. 25558849 10.1002/jbt.21683

[16] Jiang DS , Wei X , Zhang XF , et al. IRF8 suppresses pathological cardiac remodelling by inhibiting calcineurin signalling. Nat Commun. 2014;5:3303. 24526256 10.1038/ncomms4303 PMC3929801

[17] Rao BN , Rao BS , Aithal BK , Kumar MR . Radiomodifying and anticlastogenic effect of Zingerone on Swiss albino mice exposed to whole body gamma radiation. Mutat Res. 2009;677:33‐41. 19463966 10.1016/j.mrgentox.2009.05.004

[18] Xie X , Sun S , Zhong W , et al. Zingerone attenuates lipopolysaccharide‐induced acute lung injury in mice. Int Immunopharmacol. 2014;19:103‐109. 24412620 10.1016/j.intimp.2013.12.028

[19] Wu QQ , Yuan Y , Jiang XH , et al. OX40 regulates pressure overload‐induced cardiac hypertrophy and remodelling via CD4+ T‐cells. Clin Sci. 2016;130:2061‐2071. 27580926 10.1042/CS20160074

[20] Xiao Y , Yang Z , Wu QQ , et al. Cucurbitacin B protects against pressure overload induced cardiac hypertrophy. J Cell Biochem. 2017;118:3899‐3910. 28390176 10.1002/jcb.26041

[21] Wan Y , Xu L , Wang Y , Tuerdi N , Ye M , Qi R . Preventive effects of astragaloside IV and its active sapogenin cycloastragenol on cardiac fibrosis of mice by inhibiting the NLRP3 inflammasome. Eur J Pharmacol. 2018;833:545‐554. 29913124 10.1016/j.ejphar.2018.06.016

[22] Murdoch CE , Chaubey S , Zeng L , et al. Endothelial NADPH oxidase‐2 promotes interstitial cardiac fibrosis and diastolic dysfunction through proinflammatory effects and endothelial‐mesenchymal transition. J Am Coll Cardiol. 2014;63:2734‐2741. 24681145 10.1016/j.jacc.2014.02.572

[23] Zhao Y , Vanhoutte PM , Leung SW . Vascular nitric oxide: Beyond eNOS. J Pharmacol Sci. 2015;129:83‐94. 26499181 10.1016/j.jphs.2015.09.002

[24] El‐Bassossy HM , Al‐Thubiani WS , Elberry AA , et al. Zingerone alleviates the delayed ventricular repolarization and AV conduction in diabetes: Effect on cardiac fibrosis and inflammation. PLoS ONE. 2017;12:e0189074. 29206854 10.1371/journal.pone.0189074 PMC5716606

[25] Soliman AF , Anees LM , Ibrahim DM . Cardioprotective effect of zingerone against oxidative stress, inflammation, and apoptosis induced by cisplatin or gamma radiation in rats. Naunyn Schmiedebergs Arch Pharmacol. 2018;391(8):819‐832. 29736620 10.1007/s00210-018-1506-4

[26] Munzel T , Camici GG , Maack C , Bonetti NR , Fuster V , Kovacic JC . Impact of oxidative stress on the heart and vasculature: Part 2 of a 3‐Part series. J Am Coll Cardiol. 2017;70:212‐229. 28683969 10.1016/j.jacc.2017.05.035 PMC5663297

[27] Kang KW , Lee SJ , Kim SG . Molecular mechanism of nrf2 activation by oxidative stress. Antioxid Redox Signal. 2005;7:1664‐1673. 16356128 10.1089/ars.2005.7.1664

[28] Jaiswal AK . Nrf2 signaling in coordinated activation of antioxidant gene expression. Free Radic Biol Med. 2004;36:1199‐1207. 15110384 10.1016/j.freeradbiomed.2004.02.074

[29] Jones AM , Thompson C , Wylie LJ , Vanhatalo A . Dietary nitrate and physical performance. Annu Rev Nutr. 2018;38:303‐328. 30130468 10.1146/annurev-nutr-082117-051622

[30] Dimmeler S , Fleming I , Fisslthaler B , Hermann C , Busse R , Zeiher AM . Activation of nitric oxide synthase in endothelial cells by Akt‐dependent phosphorylation. Nature. 1999;399:601‐605. 10376603 10.1038/21224

[31] Chen ZP , Mitchelhill KI , Michell BJ , et al. AMP‐activated protein kinase phosphorylation of endothelial NO synthase. FEBS Lett. 1999;443:285‐289. 10025949 10.1016/s0014-5793(98)01705-0

[32] Schulz E , Jansen T , Wenzel P , Daiber A , Munzel T . Nitric oxide, tetrahydrobiopterin, oxidative stress, and endothelial dysfunction in hypertension. Antioxid Redox Signal. 2008;10:1115‐1126. 18321209 10.1089/ars.2007.1989

[33] Naseem KM . The role of nitric oxide in cardiovascular diseases. Mol Aspects Med. 2005;26:33‐65. 15722114 10.1016/j.mam.2004.09.003

[34] Deanfield JE , Halcox JP , Rabelink TJ . Endothelial function and dysfunction: testing and clinical relevance. Circulation. 2007;115:1285‐1295. 17353456 10.1161/CIRCULATIONAHA.106.652859

[35] Ramprasath T , Vasudevan V , Sasikumar S , Puhari SS , Saso L , Selvam GS . Regression of oxidative stress by targeting eNOS and Nrf2/ARE signaling: a guided drug target for cardiovascular diseases. Curr Top Med Chem. 2015;15:857‐871. 25697563 10.2174/1568026615666150220114417

[36] Khatua TN , Dinda AK , Putcha UK , Banerjee SK . Diallyl disulfide ameliorates isoproterenol induced cardiac hypertrophy activating mitochondrial biogenesis via eNOS‐Nrf2‐Tfam pathway in rats. Biochem Biophys Rep. 2016;5:77‐88. 28955809 10.1016/j.bbrep.2015.11.008 PMC5600345

[37] Wang R , Tu J , Zhang Q , et al. Genistein attenuates ischemic oxidative damage and behavioral deficits via eNOS/Nrf2/HO‐1 signaling. Hippocampus. 2013;23:634‐647. 23536494 10.1002/hipo.22126

[38] Heiss EH , Schachner D , Werner ER , Dirsch VM . Active NF‐E2‐related factor (Nrf2) contributes to keep endothelial NO synthase (eNOS) in the coupled state: role of reactive oxygen species (ROS), eNOS, and heme oxygenase (HO‐1) levels. J Biol Chem. 2009;284:31579‐31586. 19797052 10.1074/jbc.M109.009175 PMC2797228

[39] Erkens R , Suvorava T , Sutton TR , et al. Nrf2 deficiency unmasks the significance of nitric oxide synthase activity for cardioprotection. Oxid Med Cell Longev. 2018;2018:8309698. 29854098 10.1155/2018/8309698 PMC5952436

