# Supplementary material for: Zingerone attenuates aortic banding‐induced cardiac remodelling via activating the eNOS/Nrf2 pathway
Source: J Cell Mol Med. 2019 Jul 10;23(9):6466–78. doi: 10.1111/jcmm.14540 (PMC6714175; doi:10.1111/jcmm.14540)
Supplement: Supplementary file 1 [file JCMM-23-6466-s001.docx]

**Supplementary information**

**Figure S1**

**
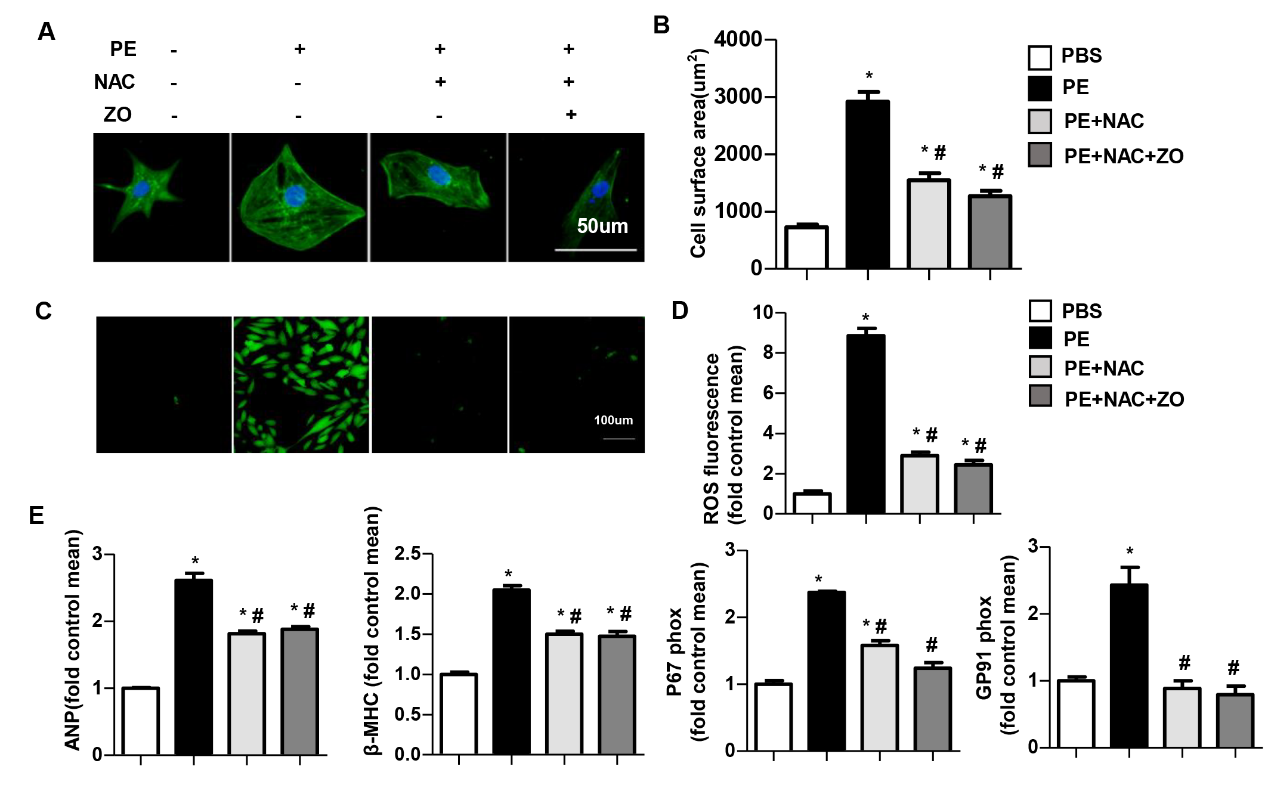
**

**Zingerone could not improve the antioxidant effect of NAC. (A)** NRCMs are treated with zingerone (250 μM) and/or NAC (2 mM), and stimulated with PE (50 μM) for 24 h. **(A-B)**The cell surface area was detected by immunofluorescence staining of α-actinin(n = 6 samples, 50+cells per group). **(A)** Representative images; **(B)** quantitative results. **(C)** Representative images of ROS detection (n = 6 samples). **(D)** Quantitative results of ROS fluorescence. **(E)** RT-PCR analysis of ANP, β-MHC, NADPH gp91 phox and NADPH p67 phox mRNA levels in each group (n = 6 samples);. *P < 0.05 versus PBS group; #P < 0.05 versus PE group.

**Figure S2**

**
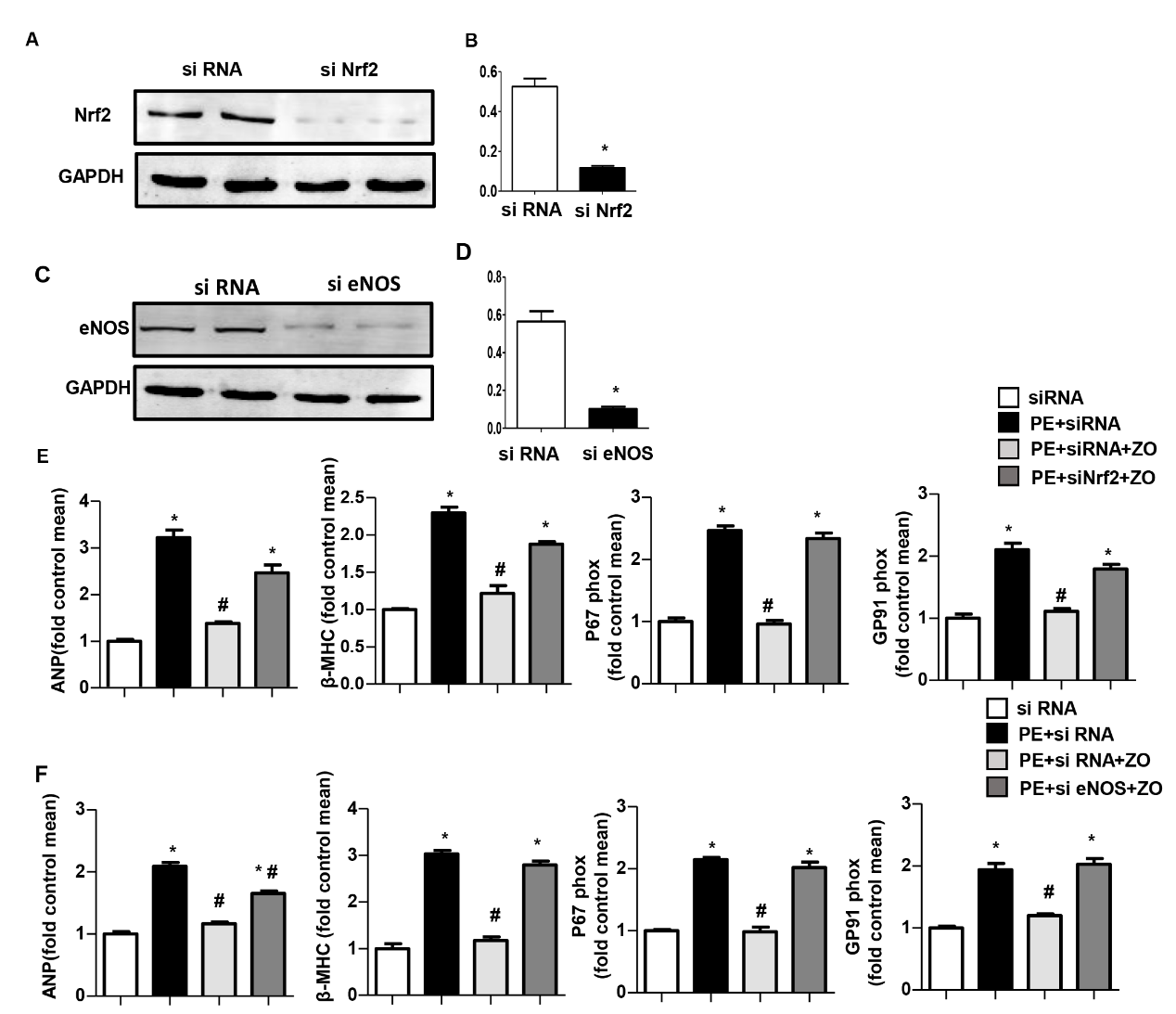
**

**Nrf2 or eNOS knock down counteracted the cardioprotection effects of Zingerone in vitro .** Western blot images**(A)** and quantitative results **(B)** of Nrf2 after NRCMs were infected with siNrf2. Western blot images**(C)** and quantitative results **(D)** of eNOS after NRCMs were infected with si eNOS. *P < 0.05 versus siRNA. **(E)** RT-PCR analysis of ANP, β-MHC, NADPH gp91 phox and NADPH p67 phox mRNA levels in each group (n = 6 samples) **(F)** RT-PCR analysis of ANP, β-MHC, NADPH gp91 phox and NADPH p67 phox mRNA levels in each group (n = 6 samples) *P < 0.05 versus siRNA group; #P < 0.05 versus siRNA+PE group.

**Figure S3**

**
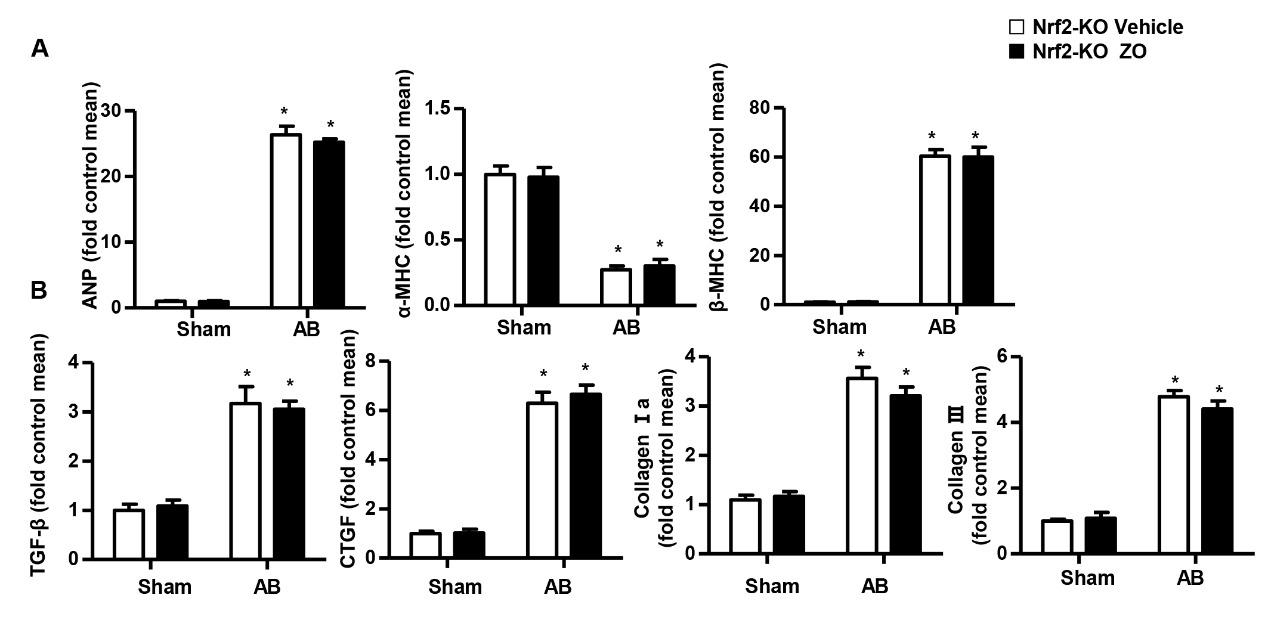
**

**
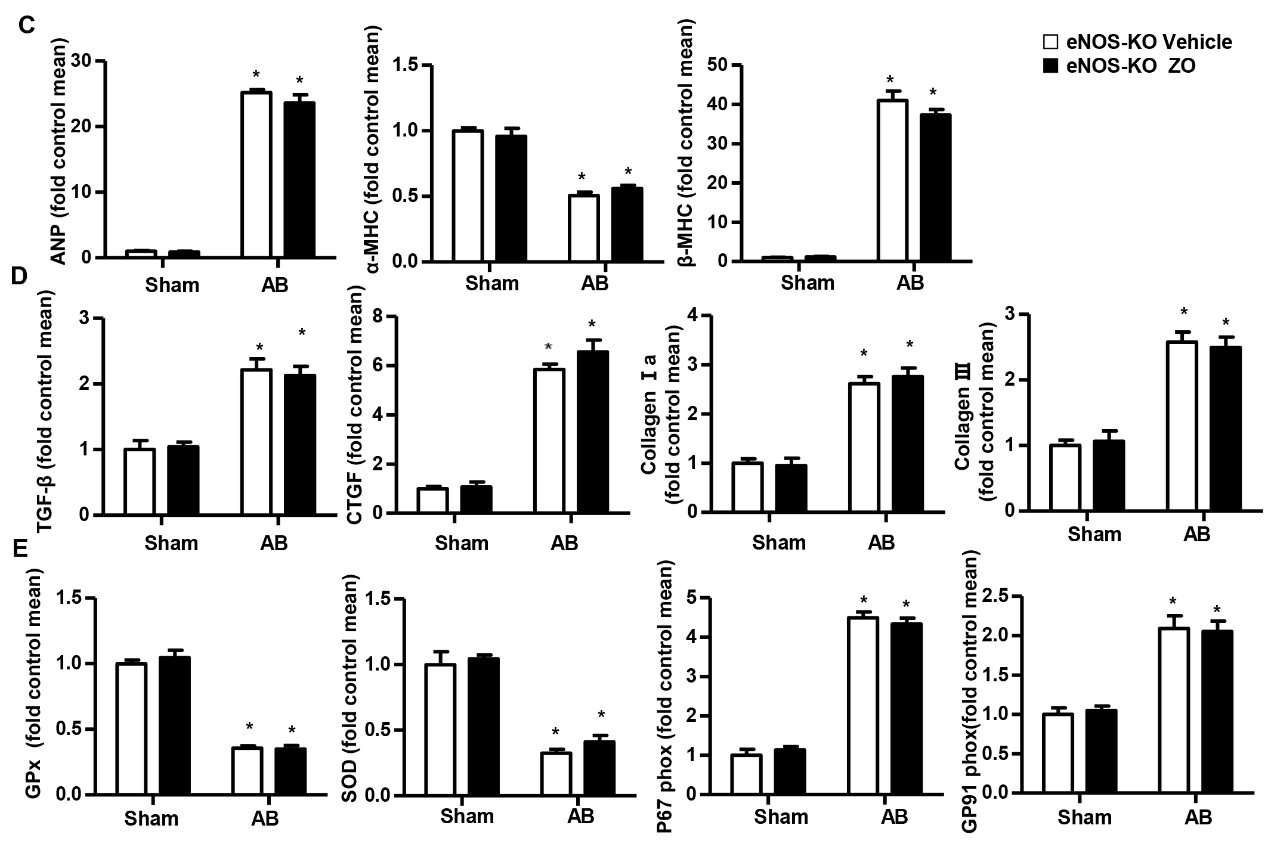
Nrf 2 or eNOS deficiency counteracted the cardioprotection effects of Zingerone in vivo .(A-B).**RT-PCR analyses of hypertrophic markers (ANP, α-MHC, β-MHC), and fibrotic markers (collagen I, collagen III, TGFβ and CTGF) in Nrf 2-KO mice of each group(n = 6).*P < 0.05 versus Nrf 2-KO vehicle-sham; #P < 0.05 versus Nrf 2-KO vehicle-AB.(C-E). RT-PCR analyses of hypertrophic markers (ANP, α-MHC, β-MHC), fibrotic markers (collagen I, collagen III, TGFβ and CTGF) and oxidative stress markers(GPx, SOD, P67 phox, GP91 phox) in eNOS-KO mice of each group(n = 6)*P < 0.05 versus eNOS-KO vehicle-sham; #P < 0.05 versus eNOS-KO vehicle-AB.

**FigureS4
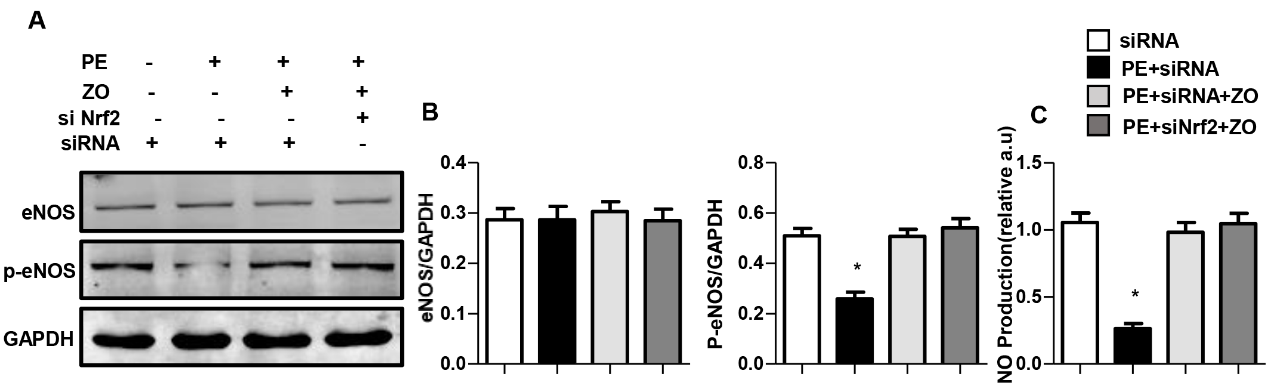
**

**Nrf2 silencing didn’t influence the expression of** **eNOS, p-eNOS (S1177) and NO-production in NRCMs.** (A-C) NRCMs were treated with zingerone (250 μM) and/or siNrf2 (20uM), and stimulated with PE (50 μM) for 24. Western blot images**(A)** and quantitative results**(B)** of eNOS and p-eNOS in each group. (n = 6 samples).; **(C)** NO production (n = 6 samples) *P < 0.05 versus si RNA group

**Table S1**

**Echocardiography and hemodynamic parameters in mice treated with zingerone or vehicle after AB surgery**

| Parameter | sham  vehicle | sham  zingerone | AB  vehicle | AB  zingerone |
| --- | --- | --- | --- | --- |
| HR | 423.37±22.26 | 402.75±15.06 | 417.37±24.61 | 421.75±35.12 |
| LVEDd(mm) | 3.72±0.25 | 3.67±0.21 | 4.90±0.28 * | 4.13±0.33 *,# |
| LVPWd(mm) | 0.76±0.05 | 0.77±0.09 | 1.27±0.15 * | 0.92±0.14 *,# |
| LVESd(mm) | 2.50±0.15 | 2.53±0.20 | 3.99±0.32 * | 3.16±0.24 *,# |
| LVEF(%) | 69.56±3.56 | 67.03±3.82 | 46.06±5.96 * | 55.07±4.90 *,# |
| LVFS(%) | 32.82±2.59 | 31.01±2.71 | 18.70±3.10 * | 23.50±2.82 *,# |
| ESP(mmHg) | 91.77±3.37 | 91.70±3.90 | 145.38±7.77 * | 143.47±7.37* |
| EDP(mmHg) | 11.55±1.05 | 11.27±1.04 | 20.80±1.62 * | 17.63±1.46*,# |
| dp/dt max(mmHg·s^-1^) | 9697±980 | 9250±757 | 5353±687 * | 6457±767*,# |
| dp/dt min(mmHg·s^-1^) | -9128±383 | -9416±651 | -6482±608 * | -7298±424*,# |

Heart rate (HR), Left ventricular ejection fraction (LVEF), left ventricular fractional shortening (LVFS), left ventricular end-diastolic diameter (LVEDd), left ventricular end-systolic diameter (LVESd)) and left ventricular end-diastolic posterior wall dimension (LVPWd),end-systolic pressure (ESP), end-diastolic pressure (EDP), minimal rate of pressure decay (dp/dt min), and maximal rate of pressure development (dp/dt max)

**Table S2**

**Echocardiography and hemodynamic parameters in Nrf2-KO mice treated with zingerone or vehicle after AB surgery**

| Parameter | sham  vehicle | sham  zingerone | AB  vehicle | AB  zingerone |
| --- | --- | --- | --- | --- |
| HR | 435.25±27.34 | 452.63±32.11 | 437.13±18.39 | 434.75±24.94 |
| LVEDd(mm) | 3.63±0.25 | 3.60±0.24 | 5.10±0.37* | 5.11±0.41* |
| LVPWd(mm) | 0.78±0.06 | 0.77±0.05 | 1.51±0.22* | 1.48±0.23* |
| LVESd(mm) | 2.49±0.25 | 2.50±0.33 | 4.33±0.29* | 4.36±0.26* |
| LVEF(%) | 66.99±8.98 | 65.06±12.60 | 37.56±14.17* | 36.16±15.59* |
| LVFS(%) | 31.33±5.87 | 30.48±8.58 | 14.95±6.38* | 14.35±6.59* |
| ESP(mmHg) | 106.00±15.51 | 100.38±12.81 | 144.75±11.04* | 145.38±14.58* |
| EDP(mmHg) | 11.26±1.24 | 10.65±1.57 | 22.03±1.82* | 21.20±2.05* |
| dp/dt max(mmHg·s^-1^) | 8696±473 | 8321±676 | 5311±670* | 5368±595* |
| dp/dt min(mmHg·s^-1^) | -8926±926 | -8613±978 | -4964±678* | -5186±967* |

Heart rate (HR), Left ventricular ejection fraction (LVEF), left ventricular fractional shortening (LVFS), left ventricular end-diastolic diameter (LVEDd), left ventricular end-systolic diameter (LVESd)) and left ventricular end-diastolic posterior wall dimension (LVPWd),end-systolic pressure (ESP), end-diastolic pressure (EDP), minimal rate of pressure decay (dp/dt min), and maximal rate of pressure development (dp/dt max).

**Table S3**

**Echocardiography and hemodynamic parameters in eNOS-KO mice treated with zingerone or vehicle after AB surgery**

| Parameter | sham  vehicle | sham  zingerone | AB  vehicle | AB  zingerone |
| --- | --- | --- | --- | --- |
| HR | 448.46±20.05 | 460.85±34.86 | 473.54±32.88 | 452.55±36.75 |
| LVEDd(mm) | 3.65±0.29 | 3.54±0.28 | 5.11±0.21* | 5.00±0.37* |
| LVPWd(mm) | 0.77±0.06 | 0.78±0.06 | 1.32±0.08* | 1.26±0.07* |
| LVESd(mm) | 2.49±0.25 | 2.44±0.32 | 4.16±0.22* | 4.12±0.21* |
| LVEF(%) | 66.34±13.62 | 65.08±15.97 | 45.13±11.05* | 43.59±6.53* |
| LVFS(%) | 31.46±8.95 | 30.84±9.92 | 18.46±5.53* | 17.48±3.17* |
| ESP(mmHg) | 110.62±12.69 | 104.83±6.67 | 140.65±2.38* | 144.84±12.71* |
| EDP(mmHg) | 10.02±1.49 | 10.10±1.37 | 24.84±3.56* | 24.68±3.55* |
| dp/dt max(mmHg·s^-1^) | 8403±799 | 8412±786 | 5191±390* | 5490±325* |
| dp/dt min(mmHg·s^-1^) | -7669±706 | -7889±803 | -4752±360* | -4864±452* |

Heart rate (HR), Left ventricular ejection fraction (LVEF), left ventricular fractional shortening (LVFS), left ventricular end-diastolic diameter (LVEDd), left ventricular end-systolic diameter (LVESd)) and left ventricular end-diastolic posterior wall dimension (LVPWd),end-systolic pressure (ESP), end-diastolic pressure (EDP), minimal rate of pressure decay (dp/dt min), and maximal rate of pressure development (dp/dt max).
